# Artificial Endoscopy and Inflammatory Bowel Disease: Welcome to the Future

**DOI:** 10.3390/jcm11030569

**Published:** 2022-01-24

**Authors:** Virginia Solitano, Alessandra Zilli, Gianluca Franchellucci, Mariangela Allocca, Gionata Fiorino, Federica Furfaro, Ferdinando D’Amico, Silvio Danese, Sameer Al Awadhi

**Affiliations:** 1 Department of Biomedical Sciences, Humanitas University, 20090 Pieve Emanuele, Italy; virginia.solitano@humanitas.it (V.S.); gianluca.franchellucci@humanitas.it (G.F.); ferdinando.damico@humanitas.it (F.D.); 2 Gastroenterology and Endoscopy, IRCCS Ospedale San Raffaele, 20132 Milan, Italy; zilli.alessandra@hsr.it (A.Z.); allocca.mariangela@hsr.it (M.A.); gionataf@gmail.com (G.F.); sdanese@hotmail.com (S.D.); 3 Università Vita-Salute San Raffaele, 20132 Milan, Italy; 4 Humanitas Clinical and Research Centre, 20089 Milan, Italy; federica.furfaro@humanitas.it; 5 Digestive Diseases Unit, Rashid Hospital, Dubai 003206, United Arab Emirates

**Keywords:** inflammatory bowel disease, artificial intelligence, endoscopy, capsule endoscopy, machine learning

## Abstract

Artificial intelligence (AI) is assuming an increasingly important and central role in several medical fields. Its application in endoscopy provides a powerful tool supporting human experiences in the detection, characterization, and classification of gastrointestinal lesions. Lately, the potential of AI technology has been emerging in the field of inflammatory bowel disease (IBD), where the current cornerstone is the treat-to-target strategy. A sensible and specific tool able to overcome human limitations, such as AI, could represent a great ally and guide precision medicine decisions. Here we reviewed the available literature on the endoscopic applications of AI in order to properly describe the current state-of-the-art and identify the research gaps in IBD at the dawn of 2022.

## 1. Introduction

Crohn’s disease (CD) and ulcerative colitis (UC) are chronic inflammatory bowel disease (IBD), with increasing incidence all around the world and a great impact on general well-being, social functioning, and utilization of healthcare resources [1,2]. The diagnosis of IBD is a daily challenge for physicians, being based on different elements such as clinical data, biochemical values, radiology, endoscopy, and histology [3]. Among them, endoscopy represents a cornerstone in the diagnosis and follow-up of CD and UC [4,5].

In the last five years, the concept of endoscopy has evolved from a traditional one to a new idea based on artificial intelligence (AI). AI is defined as any machine that has cognitive functions mimicking humans for problem solving or learning [6]. AI has already been tested in several fields of endoscopy, such as in the detection of Barrett’s esophagus [7] or the evaluation of adenoma detection rate during colonoscopy [8,9].

Attention has shifted to the potential role of AI in the field of IBD where endoscopic activity is based on several scores, such as the Mayo endoscopic subscore (MES), the Ulcerated Colitis Endoscopic Index of Severity (UCEIS), the Crohn’s Disease Endoscopic Index of Severity (CDEIS), the Lewis score, and the Capsule Endoscopy Crohn’s Disease Activity Index (CECDAI) [10,11,12,13,14]. The reason for this large number of scores lays in the need for establishing a strict definition of disease activity, thus reducing the interobserver variability and having a solid comparative analysis of different patients or studies [15]. In this context AI could be a great step forward in the research of homogeneity and reproducibility of endoscopic data. This article aims to summarize the literature data on AI endoscopic applications in the field of IBD, underlining the strengths and limitations of the currently available tools at the dawn of 2022.

## 2. What Is Artificial Intelligence and Its Current Application in Endoscopy?

AI-assisted endoscopy is based on computer algorithms that perform as human brains do [16]. They react (output) to what they receive as information (input) and what they have learned when built. The fundamental principle of this technology is “machine learning” (ML) [17].

There are many different ML methods (Table 1) and one of the most popular is the use of artificial neural networks (ANN) [18]. ANN is based on multiple interconnected layers of algorithms, which process data in a specific pattern and feed data so that the system can be trained to carry out a specific task [19]. Another diffuse ML method is the Support-vector machine (SVM), which is used for classifying data sets by creating a line or plane to separate data into distinct classes [20]. An evolution of ML is deep learning (DL): a complex, multilayer neural network architecture learns representations of data automatically by transforming the input information into multiple levels of abstractions [21,22]. An evolution of the simpler ANN is the convolution neural network (CNN), inspired by the response of human visual cortex neurons to a specific stimulus and being able to convolve the input and pass its result to the next layer [19,23].

Based on this technology, three kinds of tools have been generated to support endoscopy in each part of its activity [24,25,26]:

- Computer-aided detection (CADe), which detects gastrointestinal lesions;
- Computer-aided diagnosis (CADx), which characterizes gastrointestinal lesions;
- Computer-aided monitoring (CADm), which evaluates the procedure and the endoscopist, thus improving the quality of endoscopy.

In particular, CADe and CADx are the best developed systems with many experiences around the world demonstrating their better performance than the human eye [9,27,28,29]; for example, the GI-Genius Medtronic system reached a sensibility of 99.7% in polyps’ detection as shown by Hassan et al. [27]. The application fields of AI are expanding rapidly and IBD is the next target of this innovative technology.

## 3. AI in the Diagnosis of IBD

One of the first applications of AI has been the attempt to facilitate the diagnosis of IBD and the differential diagnosis between CD and UC. In the model of Mossotto [30], three supervised ML models were developed utilizing endoscopic data only, histological only, and combined endoscopic/histological with an accuracy of 71.0%, 76.9%, and 82.7%, respectively [30]. The model combining endoscopic and histological data was tested on a statistically independent cohort of 48 pediatric patients from the same clinic, with an accuracy of about 83.3% in patients’ classification.

Quénéhervé and colleagues [31] tried to design a model to diagnose IBD and establish differential diagnoses between CD vs. UC. They based their study on confocal laser endomicroscopy (CLE), which is an adaptation of light microscopy whereby focal laser illumination is combined with pinhole limited detection to geometrically reject out-of-focus light [32]. The authors built a score based on 14 functional and morphological parameters to perform a quantitative analysis of the mucosa called cryptometry and detect a diagnosis of IBD with a sensitivity and a specificity to near 100%. Moreover, this study reached a sensitivity of 92.3% and a specificity of 91.3% in the differential diagnosis between CD and UC.

Diagnosis of IBD can be a complex and challenging procedure due to its heterogeneous presentation. It is generally believed that making a correct diagnosis requires information on the endoscopic and histological features, together with clinical and biochemical data. AI support may be helpful in the diagnostic process by combining all suggestive features intelligently.

## 4. AI in UC, State-of-the-Art

As previously underlined, endoscopy plays a fundamental role in the diagnosis and assessment of IBD activity [5]. According to this concept, endoscopy should guarantee an exact staging of the disease and a high level of concordance between different operators. Indeed, the definition of recurrence or the assessment of remission are cornerstones in the disease management, thus guiding the next clinical or surgical decisions [33,34].

In the study of Ozawa, the authors designed a CAD system using a CNN and evaluated its performance in the identification of normal or inflamed mucosa, using a large dataset of endoscopic images from patients with UC [35]. The performance of this new tool was valuable, with areas under the receiver operating characteristic curves (AUROCs) of 0.86 and 0.98 in the identification of MES 0 (completely normal mucosa) and MES 0–1 (mucosal healing state), respectively [35]. In a similar experience from Stidham et al. [36] a CNN showed an AUROC of 0.96 in distinguishing endoscopic remission (MES = 0 or 1) from moderate to severe disease (MES = 2 or 3), with a good weighted κ agreement between the CNN and the adjudicated reference score for identifying exact MES (κ = 0.84; 95% CI, 0.83–0.86). The application of this CNN to the entirety of the colonoscopy videos had high accuracy in identifying moderate to severe disease with an AUROC of 0.97 [36].

Moreover, Gottlieb and colleagues [37] developed another recurrent neural network able to predict MES and UCEIS from entire endoscopy videos and not only from images. The system automatically selected the frame to be analyzed and scores were calculated on the colon section, showing high agreement with the human central reader score [37]. Similarly, a fully automated video analysis system was developed to assess the grade of UC activity and predicted MES in 78% of videos (κ = 0.84). In external clinical trial videos, reviewers agreed on MES in 82.8% of videos (κ = 0.78) [38]. Automated MES grading of clinical trial videos (often low resolution) correctly distinguished remission (MES = 0 or 1) vs. active disease (MES = 2 or 3) in 83.7% of videos.

Not only were automated systems able to assess endoscopic activity from still images [39], but they were also able to predict a binary version of the MES directly analyzing a raw colonoscopy video, resulting in a high level of accuracy (AUC of 0.94 for MES ≥ 1 and 0.85 for MES ≥ 2 and MES ≥ 3) [40]. Looking forward, it seems that AI can also guide real-time therapy decisions in patients with UC in clinical remission by helping to stratify the relapse risk one year after AI-assisted colonoscopy [41].

Other experiences pushed forward the application of AI in the prediction of histology. Indeed, Takenaka and colleagues [42] designed a deep neural network algorithm, defined as DNUC, based on more than 40,000 images from colonoscopies and 6000 biopsies of 875 patients prospectively collected. AI system evaluations were matched with the UCEIS score expressed for each image by three expert endoscopists and with the Geboes score determined by pathologists [43]. The DNUC revealed an accuracy of 90.9% and 92.9% in the detection of endoscopic and histological remission, respectively. In addition, Maeda et al. [44] developed a CADx system to predict persistent histological inflammation using endocytoscopy in 187 retrospectively collected patients. Endocytoscopy is one of the most valuable technologies, although it is not widely available in endoscopic departments. Providing ultra-high-resolution white light images (520x), endocytoscopy allows the so-called virtual histology or optical biopsy [45]. The results obtained by the CAD algorithm were compared with the Geboes score defined by five expert pathologists, blinded from endoscopist results. The algorithm showed a sensitivity of 74% and a specificity of 97%, with high level of reproducibility and interobserver agreement (κ value = 1).

Honzawa and colleagues [46] moved forward with the AI-application in trying to differentiate between MES 0 and MES 1 in patients with UC in clinical remission. The authors investigated the correlation among the so-called MAGIC score (Mucosal Analysis of Inflammatory Gravity by i-scan TE-c Image), MES, and histological Geboes score. Interestingly, the MAGIC score, based on the level of mean inflammation derived from all the pixels, was significantly higher in the MES 1 group than in the MES 0 group (*p* = 0.0034), with a significant correlation with histology (*p* = 0.015).

Similar to the color map of the MAGIC score, a validation study [47] elaborated an operator-independent, computer-based tool, named Red Density (RD), that determined disease activity in UC according to a redness map and vascular pattern recognition. The RD score, which is different from the previous exposed experiences as it is based on pure physics parameters, significantly correlated with the histological scoring systems (Robarts Histopathology index, r = 0.74) and with MES and UCEIS endoscopic scores with r = 0.76 and 0.74, respectively. Some weak points of this work are the monocentric experience, the small population (29 patients), and the analysis being performed only on the single picture and not on the entire colonic segment. However, this study represents an important application of AI as testified by the high level of performance. Notably, the algorithm structure does not require as much information as the CNN system due to the possibility of sequential modulation of the algorithm during the development.

Finally, a multicenter study in inactive patients with UC (PRognOstiC valuE of rEd Density in Ulcerative Colitis: PROCEED-UC; NCT04408703) is planned to assess the predictive value of the RD score for sustained clinical remission. It is plausible that the RD score might be used in the future as the first objective operator-independent endoscopic target in a treat-to-target strategy in UC. The main characteristics of the studies on endoscopic AI application in IBD are summarized in Table 2.

## 5. AI in CD, State-of-the-Art

In the field of CD, AI has been mostly applied on video capsule technology (Table 3), which has been assuming an important role both in the diagnosis and assessment of mucosa healing in the small bowel [48]. In the current European Crohn’s and Colitis Organisation (ECCO) guidelines, patients suspected to have CD but with a negative endoscopy should undergo a second level diagnostic method such as magnetic resonance imaging (MRI) or video capsule endoscopy [4]. Moreover, even in cases of normal imaging tests, such as MRI and clinical signs suspicious of small bowel CD (e.g., elevated calprotectin and/or unexplained iron deficiency anemia), video capsule endoscopy is indicated to exclude small bowel involvement [4]. However, the use of video capsules has some limitations, such as the collection of a huge amount of data and the duration of the analysis [48]. AI may overcome these barriers by selecting the frame or the part of video needed for the assessment and cutting off the time for diagnosis, thus requiring a limited amount of data to store.

The first experience was conducted about 10 years ago. Girgis et al. [49] built a system that identified the inflamed regions after a SVM training, with an accuracy of 87%, sensitivity of 93%, and specificity of 80%. Two years later, Kumar et al. [50] developed a similar system with a precision of about 90% in detecting CD lesions. Lately, several studies have been conducted for the development of systems able to automatically detect ulcers and/or aphthae and to grade mucosal damage.

A novel filtering process, called hybrid adaptive filtering (HAF), was proposed for efficient extraction of lesion-related characteristics using wireless capsule endoscopy. This system was trained on 800 images collected by 13 different patients and offered high performances in the detection of severe lesions (93.8% of accuracy, 95.2% of sensitivity, 92.4% specificity, and 92.6% of precision) [51]. The group of Klang provided two experiences in this direction [52,53]_._ The former showed an AUC of 0.99 with an accuracy ranging from 95.4% to 96.7% in classifying images into either normal mucosa or mucosa with ulcers [52]. The latter exhibited a good accuracy of 93.5% [±6.7%] in classifying strictures vs. non-strictures [53]_._

A CNN was trained to detect erosions and ulcers, demonstrating performances comparable with the activity of two expert gastroenterologists, with an AUC of 0.96 for the detection of abnormalities [54]. Interestingly, a consensus reading was used to train another CNN in automatic grading of images of CD ulcers. The resulting algorithm was tested against capsule readers, showing high accuracy in classifying severe ulcers (0.91 for grade 1 vs. grade 3 ulcers compared to 0.6 for grade 1 vs. 2) [55].

DL methods for autonomous detection and classification of CD lesions have also been applied to panenteric capsule endoscopy system that is now available allowing simultaneous investigation of the small bowel and colon. AI technology has increased the diagnostic yield and reduced interobserver variability in this integrated procedure [56,57].

Not only did AI show a high level of performance, but also a significantly faster reading with an average time of 3.5 minutes against 50 minutes for a full video of capsule endoscopy [52,58].

Some limitations of these works warrant attention. Firstly, they were made on single images and not on the entire video so that the analysis was not able to provide an overall evaluation of the validated scores for video capsule (e.g., the Lewis score). Moreover, they are retrospective cohort studies based on restricted samples of patients.

Nevertheless, all these experiences could give a great impulse to capsule endoscopy in CD. The inflammation in the proximal bowel is correlated with a worst prognosis and a higher surgical risk [59], therefore a modern method of analysis with high sensitivity and specificity is eagerly awaited in clinical practice [60].

## 6. AI for the Detection of Neoplasms in Long-Standing IBD

Given the increased risk for developing colorectal neoplasia, surveillance colonoscopy plays an important role in the management of UC [61]. The gold standard method for dysplasia surveillance is chromoendoscopy, which utilizes indigo carmine or methylene to better define the superficial gastrointestinal mucosa [62]. New endoscopic imaging technologies such as virtual chromoendoscopy, autofluorescence imaging, CLE, and endocytoscopy are now emerging, but there are only a few reports about the application of AI-assisted colonoscopy techniques for the early diagnosis of colorectal cancer [5].

**Figure 1:**
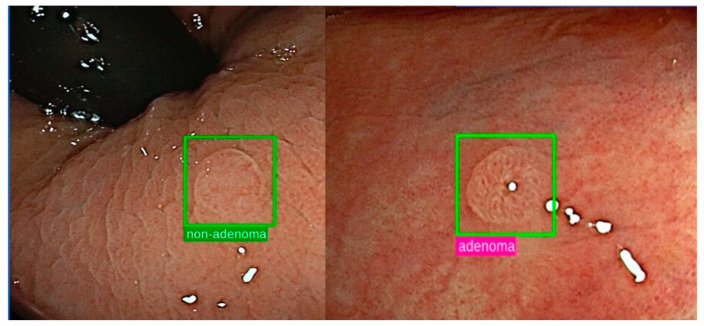
Representative images of AI-support in colorectal polyps’ characterization.

The AI capacity has been tested in the detection of colorectal neoplasia (Figure 1) but not specifically in patients with IBD.

The first experience is a case report of Maeda and colleagues [63] where the Endo-BRAIN eye system was tested for detecting dysplasia in a patient with long-standing UC. This system is able to identify colorectal lesions with high accuracy in general population [64], but in this case it proved to support endoscopists in the identification of UC-associated dysplasia, which is not always easy to detect due to its flat appearance and unclear boundaries. 

**Figure 2:**
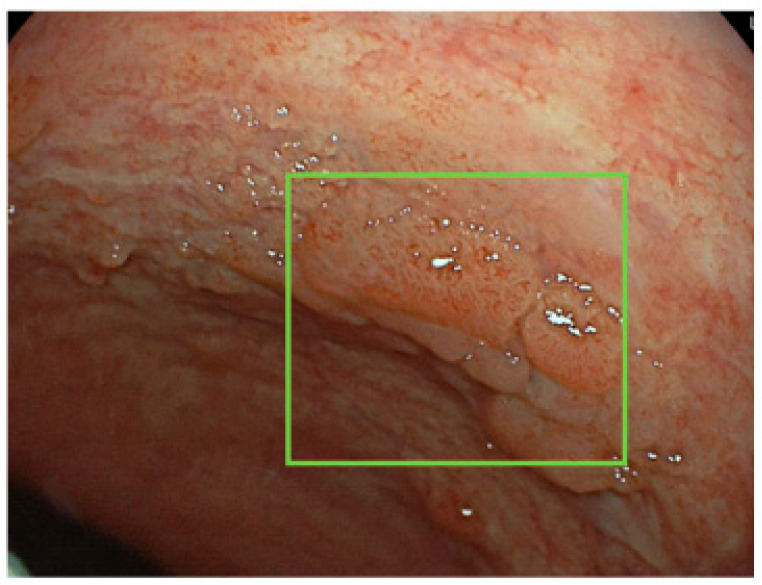
Representative image of colitis-associated dysplasia in a patient with long-standing UC.

Another example of AI-support in the detection of dysplasia was reported by Fukunaga [65]. In this case report, EndoBRAIN system helped endocitoscopy in the detection of high-grade dysplasia in a patient with long-standing UC who subsequently underwent an endoscopic submucosal dissection. To note, colitis-associated colorectal cancer may be generally difficult to diagnose due to consequences of inflammation on mucosal appearance (Figure 2) and the use of EndoBRAIN could help non-expert endoscopists to identify lesions. These experiences underline the potential and future role of AI in the colitis-associated dysplasia and neoplasia detection during IBD surveillance.

## 7. Conclusions and Future Perspectives

AI is a cornerstone revolution in endoscopy. In the field of IBD, its primary applications are providing great results in the diagnosis and staging of the disease. In a field of medicine where the current mantra is the treat-to-target strategy and where treatment directions are guided by endoscopic remission, a sensible and specific tool able to overcome human limitations could represent a great ally. High-performing diagnostic aids with low variability are useful in the detection and standardization of results and in the targets’ assessment. Moreover, if mucosal healing could be perceived as a realistic target, a concept that moves forward and takes to the extreme the previous idea is disease clearance. Even though a clear definition is still lacking, this objective includes simultaneous clinical, endoscopic, and histological remission of disease. It follows that the modern algorithms presented in the current review could help in the detection of this ambitious goal.

All the reported experiences improved the awareness about AI potential strengths and limitations. Most were nonrandomized and retrospective with small sample sizes. In addition, very limited studies were conducted to test AI support in the detection of dysplasia and neoplasia in patients with IBD. We believe these limitations should be overcome before AI becomes part of real-life practice.

In the context of AI and big data, a future perspective is the creation of algorithms for diagnosis and monitoring of IBD based not only on endoscopic, but also on clinical and histological data in order to have a complete overview of all disease features.

## Figures and Tables

**Table 1:** Algorithms involved in machine learning process.

**Supervised**	The algorithm is trained by labeling data tagged with the correct answer
**Semisupervised**	The algorithm is trained without marking the training data
**Unsupervised**	The algorithm is structured on a large amount of unlabeled data based on a small amount of labeled data

**Table 2:** Most relevant studies on endoscopic AI application in IBD.

Author (Year)	Study Design	Population	Aim	Results
Mossotto et al. (2017)	Prospective cohort study	287 paediatric IBD	To develop a ML model to classify disease subtypes	Classification accuracy with supervised ML models of 71.0%, 76.9%, and 82.7% utilizing endoscopic data only, histological only, and combined endoscopic/histological data, respectively
Quénéhervé et al. (2019)	Retrospective cohort study	23 CD patients, 27 UC patients, and 9 control patients	To test computer-based analysis of CLE images and discriminate healthy subjects vs. IBD, and UC vs. CD	Sensitivity of 100% and specificity of 100% in IBD diagnosis; sensitivity of 92% and specificity of 91% in IBD differential diagnosis
Ozawa et al. (2019)	Retrospective cohort study	26,304 colonoscopy images from a cumulative total of 841 UC patients	To test a CNN-based CAD system in identification of endoscopic inflammation severity	AUROCs of 0.86 and 0.98 to identify MES 0 and 0–1, respectively
Stidham et al. (2019)	Retrospective cohort study	16,514 images from 3082 UC patients	To test DL models in grading endoscopic severity of UC	AUROCs of 0.96, PPV of 0.87, sensitivity of 83.0%, specificity of 96.0%, and NPV of 0.94 in distinguishing endoscopic remission from MES 2–3
Gottlieb et al. (2021)	Phase II randomized controlled study	249 UC patients	To test a recurrent neural network model in predicting MES and UCEIS from individual full-length endoscopy videos	Excellent agreement metric with a QWK of 0.84 for MES and 0.85 for UCEIS
Yao et al. (2021)	Phase II randomized controlled study	315 videos from 157 UC patients	To test a fully automated video analysis system for grading endoscopic disease	Excellent performance with a sensitivity of 0.90 and specificity of 0.87; correct prediction of MES in 78% of videos (k = 0.84)
Bhambhani et al. (2021)	Retrospective cohort study	777 endoscopic images from 777 UC patients	To test a DL models in the automated grading of each individual MES	AUC of 0.89, 0.8, and 0.96 for classification of MES 1, 2, and 3, respectively; overall accuracy of 77.2%
Becker et al. (2021)	Prospective cohort study	1672 videos from 1105 UC patients	To test a DL–based system on raw endoscopic videos	AUC of 0.84 for MES ≥ 1, 0.85 for MES ≥ 2 and 0.85 for MES ≥ 3
Maeda et al. (2021)	Prospective cohort study	145 UC patients	To test AI in stratifying the relapse risk of patients in clinical remission	Relapse rate significantly higher in the AI-active group than in the AI-healing group (28.4% vs. 4.9%, *p* < 0.001)
Takenaka et al. (2020)	Prospective cohort study	40,758 images of colonoscopies and 6885 biopsy results from 2012 UC patients	To test a DNN system based on endoscopic images of UC for predicting endoscopic and histological remission	Accuracy of 90.1% and κ coefficient of 0.798 for endoscopic remission; accuracy of 92.9%and κ coefficient of 0.85 for histological remission
Maeda et al. (2019)	Retrospective cohort study	187 UC patients	To test a CAD system in predicting persistent histologic inflammation using EC	Sensitivity, specificity, and accuracy of 74%, 97%, and 91%, respectively; κ =1
Honzawa et al. 2019	Retrospective cohort study	52 UC patients in clinical remission	To test a new endoscopic imaging system using the iscan TE-c (MAGIC score) to quantify mucosal inflammation in patients with quiescent UC	MAGIC score significantly higher in the MES 1 than in the MES 0 group (*p* = 0.0034); MAGIC score significantly correlated with the Geboes score (*p* = 0.015)
Bossuyt et al. (2020)	Prospective cohort study	29 UC patients and 6 controls	To test a RD algorithm based on channel of the red-green-blue pixel values and pattern recognition from endoscopic images	Good correlation between RD and RHI (r = 0.74, *p* < 0.0001), MES (r = 0.76, *p* < 0.0001), and UCEIS (r = 0.74, *p* < 0.0001)

**Abbreviations:** AUC: area under the curve; AUROC: areas under the receiver operating characteristic curve; CAD: computer-assisted diagnosis; CD: Crohn’s disease; CLE: confocal laser endomicroscopy; CNN: convolution neural network; DL: deep learning; DNN: deep neural network; IBD: inflammatory bowel disease; MAGIC: Mucosal Analysis of Inflammatory Gravity by i-scan TE-c Image; MES: Mayo endoscopic subscore; ML: machine learning; NPV: negative predictive value; PPV: positive predictive value; QWK: quadratic weighted kappa, RD: red density; RHI: Robarts Histopathology index; UC: ulcerative colitis, UCEIS: Ulcerative Colitis Endoscopic Index of Severity.

**Table 3:** Most relevant studies on video capsule AI application in CD.

Author (Year)	Study Design	Population	Aim	Results
Girgis et al. (2010)	Retrospective cohort study	47 videos from 29 CD, 17 control, 1 celiac patient	To test a system able to detect inflammation among the thousands of images acquired by the WCE	Total accuracy, specificity, and sensitivity of 87%, 93%, and 80%, respectively
Kumar et al. (2012)	Retrospective cohort study	47 videos, 30 of which contained CD lesions	To test a supervised classification for CD lesions and for quantitative assessment of lesion severity	Good precision (>90% for lesion detection) and recall (>90%) for lesions of varying severity
Charisis et al. (2016)	Retrospective cohort study	800-image database from 13 CD patients	To test HAF-DLac approach for automated lesion detection	Accuracy, sensitivity, specificity, and precision of 93.8%, 95.2%, 92.4%, and 92.6%, respectively
Klang et al. (2020)	Retrospective cohort study	17,640 CE images from 49 CD patients	To test a CNN in classifying images into either normal mucosa or mucosal ulcers	AUC of 0.99 and accuracy ranging from 95.4% to 96.7%
Klang et al. (2021)	Retrospective cohort study	27,892 CE images	To test a DLN for detecting CE images of strictures	For classification of strictures vs. nonstrictures, average accuracy of 93.5% (±6.7%)
Barash et al. (2021)	Retrospective cohort study	17,640 CE images from 49 CD patients	To test a CNN in automatically grading images of ulcers and compare the resulting algorithm with a consensus reading	Algorithm accuracy of 0.91 for grade 1 vs. grade 3 ulcers, of 0.78 for grade 2 vs. grade 3, and of 0.62 for grade 1 vs. grade 2
Majtner et al. (2021)	Retrospective cohort study	7744 images from 38 CD patients (small bowel 4972, colon 2772)	To test the ability of a DL framework to detect lesions with panenteric capsule endoscopy	Diagnostic accuracy of 98.5% for small bowel and 98.1% for colon
Ferreira JPS et al. (2021)	Retrospective cohort study	8085 images	To develop and validate a CNN for ulcer and erosion detection using panenteric capsule endoscopy images	Model sensitivity, specificity, precision, and accuracy of 90.0%, 96.0%, 97.1%, and 92.4%, respectively

**Abbreviations:** AUC: area under the curve; CD: Crohn’s Disease; CE: capsule endoscopy; CNN: convolutional neural network; DL: deep learning; DLac: differential lacunarity; DLN: deep learning network; HAF: hybrid adaptive filtering; WCE: wireless capsule endoscopy.
